# Electrostatic and Functional Analysis of the Seven-Bladed WD β-Propellers

**DOI:** 10.4137/ebo.s743

**Published:** 2008-06-13

**Authors:** Najl V. Valeyev, A. Kristina Downing, John Sondek, Charlotte Deane

**Affiliations:** 1 Department of Biochemistry, University of Oxford, South Parks Road, Oxford, OX1 3QU, U.K; 2 Department of Pharmacology, University of North Carolina, 27599-7365, U.S.A; 3 Department of Statistics, University of Oxford, 1 South Parks Road, Oxford, OX1 3TG, U.K

**Keywords:** β-propeller, electrostatics, evolutionary trace, protein clustering, WD protein

## Abstract

β-propeller domains composed of WD repeats are highly ubiquitous and typically used as multi-site docking platforms to coordinate and integrate the activities of groups of proteins. Here, we have used extensive homology modelling of the WD40-repeat family of seven-bladed β-propellers coupled with subsequent structural classification and clustering of these models to define subfamilies of β-propellers with common structural, and probable, functional characteristics. We show that it is possible to assign seven-bladed WD β-propeller proteins into functionally different groups based on the information gained from homology modelling. We examine general structural diversity within the WD40-repeat family of seven-bladed β-propellers and demonstrate that seven-bladed β-propellers composed of WD-repeats are structurally distinct from other seven-bladed β-propellers. We further provide some insights into the multifunctional diversity of the seven-bladed WD β-propeller surfaces. This report once again reinforces the importance of structural data and the usefulness of homology models in functional classification.

## Introduction

**Figure 1:**
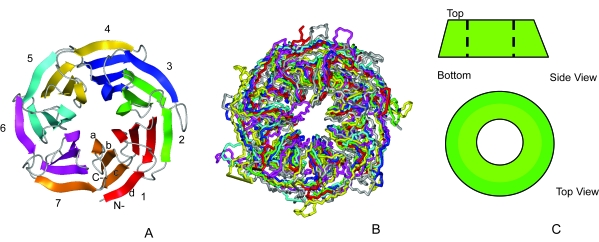
Seven-bladed β-propeller proteins. A. Seven-bladed β-propeller (1got) coloured according to the WD repeats. B. Backbone representation of the superimposed WD repeat template structures (1erj—red, 1got—green, 1gxr—blue, 1k8k—yellow, 1pgu—magenta, 1p22—cyan, 1sq9—grey). C. A cartoon representation of a β-propeller. The structure is different in radius on the “top” and “bottom”. The narrower side is defined as the “top” region in this report. Both A and B show the top surface. The strands *a*-*d* form seven blades of the propeller structure.

WD40 repeat-like proteins form a structurally conserved superfamily and are part of the β-propeller fold. The WD repeat was first characterized by Van der Voorn and Ploegh (van der Voorn and Ploegh, 1992). It is a motif containing about 40–60 amino acids ending in a tryptophan and aspartic acid (WD). Multiple X-ray structures of this repeat revealed that it represents a blade formed from a four-stranded antiparallel β sheet. These blades are arranged radially forming a β-propeller structure and this propeller is structurally conserved despite high divergence in sequence amongst β-propeller proteins (Paoli, 2001) (Fig. 1). Perhaps surprisingly, in general a single WD repeat forms parts of two blades within a propeller.

The inner strand α of each blade is situated in the centre of the propeller and strand *d* (Fig. 1A) forms the “outer” side of the propeller. Superimposition and analysis of the structures by various authors suggested that strands *a*–*c* are generally conserved and most variability is observed in strand *d* and in the connecting segments between blades (Murzin, 1992). The innermost strands form a channel which has a conical shape. In most of the propeller proteins the joining of the N- and C-termini to enclose the propeller circle is achieved by the pairing of strand *d* from the N-terminus with three strands (*a*–*c*) from the C-terminus in the same blade (Fig. 1A).

Murzin (Murzin, 1992) developed a model for the propeller assembly of β-sheets and carried out an analysis on the multi-sheet packing of the blades into propeller structures. His calculations showed that four is the only possible number of strands in a blade and that any additional strands would generate unacceptably close packing of the strands. This geometrical model also predicted that an ideal seven-bladed β-propeller satisfies average β-geometry best. He found that the seven-fold symmetry has no strong sequence constraints and is preferable to six- or eight-fold blades for assembly.

The seven-bladed β-propellers in SCOP (Murzin et al. 1995) are classified into several superfamilies, the one of interest to this work is the WD40-repeat like superfamily. This superfamily is further split into two families: the WD40-repeat, the family we shall study further, and the cell cycle arrest protein BUB3. This second family is thought by the SCOP authors to be possibly related to the WD-repeat family, but both sequence similarity between the blades and the WD40 repeat signature are very weak. The seven-bladed protein domains found in the WD40-repeat family are listed in Table 1 with annotated functions and available PDB (1got (Lambright et al. 1996), 1erj (Sprague et al. 2000), 1gxr (Pickles et al. 2002), 1k8k (Robinson et al. 2001), 1p22 (Wu et al. 2003), 1pgu (Voegtli et al. 2003), 1sq9 (Madrona and Wilson, 2004)) structures. A superposition of the protein domains for the seven-bladed WD40-repeat β-propeller family is shown in Figure 1B.

The WD motif has been extensively studied in sequence space, over 900 repeats have been aligned in order to refine the pattern (Smith et al. 1999). The relationship between amino acid sequence and function for WD repeat containing proteins has been addressed by Yu et al. (Yu et al. 2000). This analysis relies entirely on sequence similarity and does not use information from the 3D (three-dimensional) structures of the proteins. The outcome of the Yu et al. (Yu et al. 2000) study was the separation of 278 protein sequences into different groups, with each group possessing potentially unique functions. Sequences with unassigned functions were predicted to have the same function as other proteins in the same group. A second study on functional annotation of WD proteins by Yu et al. (Yu et al. 2000) defined 32 functional groups amongst the WD proteins with various numbers of blades. This result was based on the assumption that surface similarity defines a common function for WD repeat proteins (Smith et al. 1999). Only three seven-bladed β-propeller families G protein, Groucho, and Tup1 were identified amongst those functional clusters.

In this report we use structural rather than just sequence information to classify seven-bladed WD β-propellers. While the sequence data certainly gives some information, 3D structures are usually required to gain detailed biological and functional characterization (Chakravarty and Sanchez, 2004). The value gained from the comparative modelling of proteins over sequence alone has been demonstrated previously (Chakravarty and Sanchez, 2004; Chakravarty et al. 2005). Here we carried out large-scale modelling of the seven-bladed WD β-propellers followed by comparative structural analyses using the evolutionary trace (ET) method (Lichtarge et al. 1996), examination of surface cavities (Tsodikov et al. 2002) and electrostatics analyses (Blomberg et al. 1999).

ET uses a phylogenetic tree of a protein family and identifies evolutionary conserved residues in branches of the tree. Many studies report that residues predicted by ET to be evolutionary privileged significantly overlap with functional sites (Lichtarge et al. 1996; Lichtarge et al. 1996; Lichtarge et al. 1997; Lichtarge and Sowa, 2002; Lichtarge et al. 2002; Lichtarge et al. 2003). It has also been shown that such positions form statistically significant clusters (Madabushi et al. 2002). We have applied this technique to explore the functional surface interface in combination with experimentally determined key surface residues found to be important for some seven-bladed WD β-propeller families.

Another important factor determining how proteins function is their surface cavities or clefts. It has been reported that small surface cavities could be important for binding of allosteric effectors (DesJarlais et al. 1988). It has also been proposed (Kuntz et al. 1982; DesJarlais et al. 1988) that enzymatic active sites are usually situated in large surface clefts. Rigorous analysis of the importance of the cleft size for protein-protein interactions has been carried out previously (Laskowski et al. 1996).

Electrostatic potential is a protein property which can only be examined with structural knowledge. Recently, several studies have used electrostatic surface potential as a tool for predicting protein functions and for predicting functionally similar groups in protein families. Livesay et al. looked at the electrostatics near the active site of 54 experimentally determined structures of four enzyme families and one superfamily (Livesay et al. 2003). In another example, Botti et al. studied cholinesterases and neural cell-adhesion proteins (12 experimentally determined structures in total) (Botti et al. 1998). The authors showed that in the absence of any sequence similarity these protein families have a common electrostatic motif at the catalytic site. Homology modelling of 104 Pleckstrin Homology (PH) domains (Blomberg and Nilges 1997) followed by electrostatic analysis (Blomberg et al. 1999) revealed a high degree of functional conservation across the superfamily. Electrostatic calculations have also been used to analyze RNAs recognition and stabilization properties (Chin et al. 1999). Chin et al. have calculated electrostatic properties of some structures of RNA molecules from Protein Data Bank and Nucleic Acid Database (Chin et al. 1999). The authors found that the sites with unusual electrostatic features correspond to the functionally important regions. Large scale homology modelling work has also been done on complement control protein (CCP) modules (Soares et al. 2005). In total, 136 models of CCP domains were generated and subjected to multiple methods of surface characterization. In particular, it was found that assignments to clusters based on the surface properties differed from assignments based on sequence similarity. This result was suggested to reflect a particular role of surface exposed residues crucial for protein-protein interactions. The applied surface characterization methods were indeed able to pinpoint functional sites within CCP modules. In the work described here, we carry out functional protein characterization on a larger scale. We have created a large set of unique homology models (~700) from which the smaller subset of models (~170) was used to carry out the functional classification of β-propellers containing seven WD repeats.

We have performed large-scale modelling of seven bladed WD β-propeller proteins. The models were electrostatically characterized by the PIPSA program (Blomberg et al. 1999) with default parameters, and then analysed using the statistical package R (http://www.r-project.org/). We show that by using the surface electrostatics as a criterion, the members of the seven-bladed β-propeller WD40-repeat family can be classified into functional clusters. This classification is possible given high accuracy homology models. If the sequence identity between the template and the modelled sequence is too low, the classification becomes less reliable due to the increasing error. We argue that this method can predict biological role or function of uncharacterized protein sequences. The electrostatics-based classification differs from clusters based on sequence similarity. This result may suggest an important role of protein surface charge distribution for functional interactions. We also calculated the evolutionary trace (Lichtarge et al. 1996), and surface cavities (Tsodikov et al. 2002) of the seven-bladed WD40-repeat β-propeller proteins. The combination of residues predicted by these methods together with experimentally identified residues are given in Table 1. These residues are possibly important in the WD40 interactions with binding partners. It is clear from experimental data (Table 1) that seven-bladed WD β-propeller proteins have a wide variety of functions. This diversity is clearly shown to arise from multiple functional sites on the surface rather that adoption of a single site. Furthermore, in those subfamilies not yet experimentally characterized, these techniques also predict a multifunctional interface.

## Materials and Methods

### PSI-PLAST search and modelling procedures

A set of template structures for the seven-bladed WD40 family were extracted from the SCOP v1.69 database (Murzin et al. 1995). A BLAST (basic local alignment search tool) (http://www.ncbi.nlm.nih.gov) (Lipman et al. 1989; Altschul et al. 1997) search using each template as a query gave a representative sequence subset for each subfamily defined in SCOP under WD40-repeat family section. BLAST was run with an E-value inclusion threshold equal to 0.005. Since the object of this study was to analyse the structure-function relationship of seven-bladed WD β-propellers, all BLAST searches were performed using only propeller domains and extraneous protein sequence was not included. The sequences collected by BLAST were aligned against the experimental structures using ClustalW (Thompson et al. 1994). All unique hits from BLAST searches with greater than 20% sequence identity to the queries, were used to produce homology models with Modeller (Sali and Blundell, 1993), resulting in about ~1400 unique models. This entire process was fully automated. Each model was then inspected in order to identify those which were poorly built resulting from incorrect automated alignment or other errors. Incorrect models (structures with severe distortions from the propeller shape) were excluded from further analysis. In total 688 correctly built models were analysed. A smaller subset of models (166) with sequence identity cut-offs at 90% for 1got, 1gxr, 1k8k, 1pgu, 1p22 families, at 70% for the 1erj family, and at 40% for the 1sq9 family was used for testing in this study. The models of sequences meeting the cut-off criteria to the experimental structures from WD40-repeat family as defined in SCOP v1.69 database were built using Modeller program. To carry out the electrostatic analysis, the models were superimposed using a script written in Perl and the CE program (Shindyalov and Bourne, 1998).

### Structural comparison

The β-propeller parts of all seven-bladed β-propeller containing structures defined in the SCOP database were extracted. Structural comparison of all seven-bladed β-propellers was performed automatically by pairwise superimposition using the CE program based on the C_α_ atom positions. The RMSD and sequence identity and the sequence similarity (using the BLOSUM62 matrix (Henikoff and Henikoff, 1992)) were calculated for each superimposition.

The geometrical characterization of the seven-bladed β-propeller domains was performed using a simple C program. This program uses the coordinates of a given point on the outer blade of the superimposed β-propellers as an input. These points (the middle of the outer strand) were chosen by visual inspection of each representative structure. Vectors are calculated from the centre of mass to the chosen points. These vectors are characteristic for each structure. (Fig. 2 in Supplementary Material).

### Electrostatics

The structurally superimposed WD propellers were used as input for PIPSA. This program computes the similarity indexes between pairs of proteins based on the monopole and dipole terms of the molecule’s electrostatics. The Hodgkin index is calculated to measure the similarity between two molecular potentials. This parameter encompasses the differences in sign, magnitude, and spatial behaviour of the potentials. The program calculates pairwise electrostatic indexes of multiple structures allowing comparisons of large datasets and subsequent clustering analysis. The calculated electrostatic indexes can range from −1 to 1. If the Hodgkin index equals 1, the two potentials are identical, if it equals 0 the potentials are fully uncorrelated, and −1, the potentials are anticorrelated. These 3D electrostatic descriptions of the proteins were clustered using Agnes (Struyf et al. 1997) as coded in the R statistical package.

### Alternative characterization methods

The ET method was used to identify residues conserved throughout evolution in each seven-bladed WD40-repeat β-propeller subfamily. ET identifies conserved residues in sets of sequences which are in a specific region of the phylogenetic tree. If these residues are in statistically significant structural clusters, they are identified by ET and can be used for functional sites prediction. The sets of sequences are identified using “cut-off” lines. These are drawn on the phylogenetic tree and called “partitions”. For the ET analysis presented here, ten partitions were used with the 10th partition encompassing the most class-specific residues. Residues, predicted to be conserved within each partition are divided into four categories. Exposed class specific residues (from partition 10) predicted by the method were mapped onto the surface of the representative experimental structures (1got (Lambright et al. 1996); 1erj (Sprague et al. 2000); 1gxr (Pickles et al. 2002); 1k8k (Robinson et al. 2001); 1p22 (Wu et al. 2003); 1pgu (Voegtli et al. 2003); 1sq9 (Madrona and Wilson, 2004)).

The program Surface Racer (Tsodikov et al. 2002) was used to perform protein surface curvature analysis and to identify potential cavities, which serve as additional hints for functional regions. The probe radius was chosen to be slightly larger than a water molecule radius and was equal to 1.5 . The experimentally determined functional residues were compared with the ET and the surface curvature program predictions.

## Results and Discussion

### WD proteins are a structurally conserved subfamily

In order to investigate the WD40-repeat family which is a part of the seven-bladed β-propeller fold we focused on the ‘global’ geometry of the fold and have examined the arrangement of blades within the ‘plane’ of the β-propeller. There are potentially two geometrical “regions” that one could analyse in the β-propeller type structures. One geometrically distinct part is the relatively rigid protein core composed of strands arranged into blades. The question to ask is whether or not these strands are arranged differently in different subfamilies within the WD40-repeat family. Another potentially interesting question is whether there is any difference between subfamilies in the inherently flexible regions of the β-propellers, such as loops connecting the blades. There are of course a variety of geometrical parameters that one can use to characterize symmetrical β-propeller structures. For example, one could look at the characteristic distances between corresponding strands in the neighbouring blades to see if they are conserved or varied between subfamilies. Another potential option is to measure radius and height of the β-propellers. We calculated vectors (for representative experimental structures 1got (Lambright et al. 1996); 1erj (Sprague et al. 2000); 1gxr (Pickles et al. 2002); 1k8k (Robinson et al. 2001); 1p22 (Wu et al. 2003); 1pgu (Voegtli et al. 2003); 1sq9 (Madrona and Wilson, 2004)) from the centre of mass to a defined point on the outer side of each blade, this simple parameter contains information about the radius and implicitly describes the height of the structures as well as angular distribution of the individual blades. Given that the blades are composed of four anti-parallel beta strands, we used the middle of the outer strand as the characteristic measure of each blade. This approach allows analysis of how symmetrically the blades are arranged within each subfamily. The spatial distribution of the ‘middle point’ coordinates was mapped on the superimposed seven-bladed β-propeller, representative for different subfamilies (representative experimental structures listed in Material and Methods) (Fig. 2 in Supplemental Material). This analysis demonstrated that the spatial organization of the blades (the actual propeller) appears quite conserved across all the seven-bladed subfamilies within WD40-repeat family. This result distinguishes the core part of the β-propellers from the flexible loops, in which some variability has been observed between different subfamilies.

**Figure 2:**
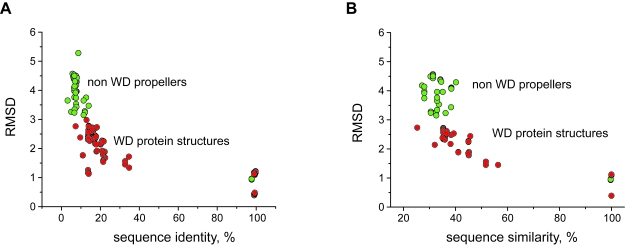
The variation of RMSDs and sequence identity between seven-bladed β-propeller proteins. The data points for the comparison between WD-WD proteins are coloured as red, and the green circles represent superimpositions between two non-WD proteins as well as between WD and non-WD proteins. The data shows clear structural conservation of the WD40 superfamily amongst all other seven-bladed β-propellers. RMSD has been calculated based on C_α_ atom positions.

We next examined more general structural diversity within the WD40-repeat family of seven-bladed β-propellers. For example, it has not been previously established that seven-bladed β-propellers composed of WD-repeats are structurally distinct from other seven-bladed β-propellers and we addressed this point by determining the structurally similarity between the two sets of domains. High structural similarity was found within the WD40-repeat protein family which was not found in any other seven-bladed β-propeller superfamily. Figure 2 shows a comparison of percentage sequence identity (A) and sequence similarity (B) with the corresponding RMSDs, for all experimentally determined seven-bladed β-propeller domains (some representative structures are shown in Figure 1 of the Supplementary Material). Overall, the pairwise comparison of WD-repeat containing domains produces significantly lower RMSDs relative to identical comparisons of pairs of domains from other β-propeller classes or between these classes and WD-repeat containing domains. This observation is rather intriguing, because there are no obvious factors explaining why the WD sequence motif would have such a high impact on the structure. In fact, the sequence identity (and the sequence similarity) amongst many of the WD proteins is as low as the sequence identity across non-WD proteins.

### Homology models of WD β-propellers

To characterize the WD40-repeat protein family in more detail, we built models of sequences with 20% or greater sequence identity to the representative structures. The models were inspected for any modelling artefacts and well built models were used in the later analysis. It was clear that with decreasing sequence identity to the template, the quality of modelling performance decreased. A set of highly similar models (166) were used to fully explore the potential of the value gained from homology modelling.

### Surface electrostatics of seven bladed WD structures is conserved

The high structural conservation of the WD propellers despite their low sequence identity allows the building of homologous models of reasonable quality in an automated manner. The WD pattern observed in this family also improves the homology model building by allowing a high degree of confidence in the alignment of sequence with the template structure.

We studied which β-propeller protein structure properties might reveal differences in function between structurally similar subfamilies. Amongst various protein surface properties important for protein-protein interactions, such as cavities, hydrophobic residues, specific interaction residue pockets, electrostatics is the one we identified to have high potential for functional protein classification. It is known that electrostatics plays an important role in molecular interactions (Honig and Nicholls, 1995). Surface electrostatic patches on proteins affect the specificity of protein-ligand or protein-protein interactions (Honig and Nicholls, 1995). Electrostatic potentials generated by molecules have a variety of different characteristic surface features that can only be calculated when a structure is known. The surface electrostatic pattern of any charged protein creates either attractive or repulsive forces. These, inevitably, make certain protein-protein interactions more or less favourable. We have used our models of the seven-bladed β-propeller subfamilies to assess whether electrostatics is applicable in this particular case. As we use protein models rather than experimental structures, one can possibly expect some inaccuracy.

**Figure 3:**
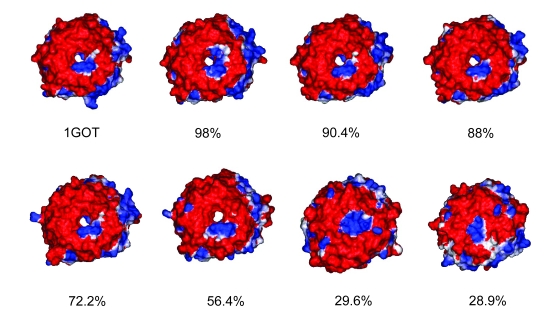
The surface of G-beta protein and its homology models coloured by their electrostatic potential. The percentages given below each structure are the sequence identity between the homology model and the structural template (1GOT). The negative charge is shown as red, positive charge is coloured blue. The conservation of the electrostatic surface in the models even those with relatively low sequence identity, is striking. All structures show the propeller’s top surfaces.

**Figure 4:**
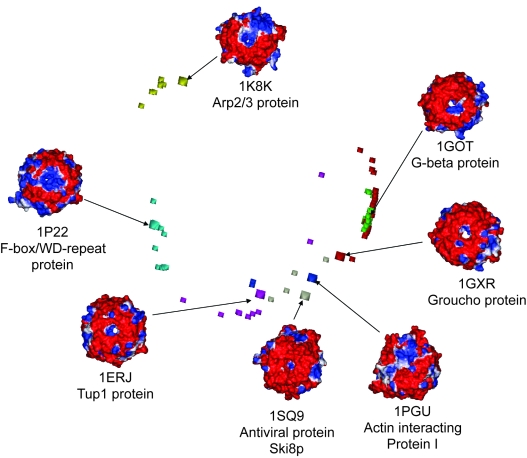
Electrostatic based grouping of the seven bladed WD repeat β-propellers. Two dimensional plot of three dimentional data is shown for PIPSA calculated electrostatic indexes of seven experimentally determined WD repeat proteins from different families and homology models. The squares represent experimentally defined structures (1erj—magenta, 1got—green, 1gxr—red, 1k8k—yellow, 1pgu—blue, 1p22—cyan, 1sq9 – grey). Electrostatics are shown for the top surfaces of proteins.

A random subset of WD protein homology models (ten from each subfamily) were selected and used initially to analyse surface electrostatics. From this we identified that surface electrostatics were highly conserved within a subfamily and different between WD40-repeat subfamilies. Figure 3 shows the surface electrostatics of the homology models in the G-beta protein subfamily. The surface pattern remains similar even though the sequence identity to the experimental template (1GOT) decreases to around 30%. This finding indicates that it may be possible to classify protein function based on the value gained from modelling and using electrostatics as a criterion. The difference in surface between the subfamilies of the WD domains can be seen in Figure 4.

From the visual inspection of the electrostatic potentials it is evident that each WD subfamily has a unique isopotential surface (Fig. 4). To measure quantitatively the degree of similarity of electrostatic properties between the subfamilies, we calculated similarity indexes between all the models using the PIPSA program. Figure 4 shows the electrostatics-based grouping for the smaller set of 166 models. It follows from this picture that WD subfamilies differ from each other despite the striking structural similarity within the WD40-repeat family. Based on these results one may argue that it is possible to predict functions of uncharacterized protein sequences (with no defined function or solved structure) based on electrostatic clustering. For example, sequences that cluster with Tup1 are transcription factors, the ones clustering with Gβ can be involved in signal transduction. However, these predictions are prone to some potential errors due to: i) homology models represent protein structures with limited accuracy; ii) electrostatic analysis is still a new approach for protein function classification.

**Figure 5:**
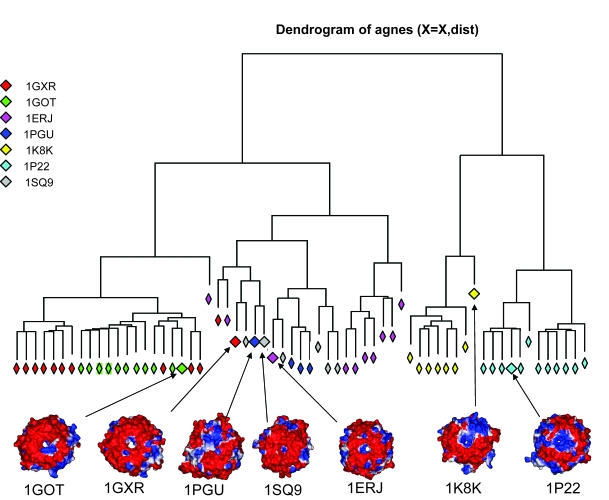
Electrostatic based clustering of the seven bladed WD repeat β-propellers. The different seven-bladed WD β-propeller sub-familes are clearly in separate clusters. Five (1GOT, 1SQ9, 1ERJ, 1K8K, 1P22) out of seven subfamilies appear to have good clustering in electrostatic space. The partial overlap of 1GXR and 1PGU families indicates that their electrostatic potentials are similar. Electrostatics are shown for the top surfaces of proteins.

While one can clearly observe certain electrostatics based clustering on Figure 4, it is important to be able to quantify this result using a formal criterion. The observed electrostatic grouping was clustered in the R statistical package. All 166 models were clustered but in order to visualize electrostatics-based separation, we also performed this analysis on a representative subset, of up to 10 randomly picked models from each subfamily (Fig. 5). The degree of clustering shown in Figure 5 is very similar to that shown by all 166 models. The clustering shows separate branches that reasonably distinguish models from different subfamilies. Homology models of five out of the seven subfamilies are clustered on the same branch as their representative experimental structure, whereas models of two subfamilies (1GXR and 1PGU) do not cluster as tightly. This might be due to the limited accuracy of comparative models as well as similar electrostatics of different subfamilies which leads to the ‘mixing’ of subfamilies in the electrostatic space. These data suggest that electrostatics provides sufficient functional distinction and can potentially have predictive power. The applied method can suggest whether or not a structure built from an uncharacterized sequence belongs to a particular functional group. It, therefore, appears possible to classify protein sequences based on the information gained from homology modelling and electrostatics. Table 1 in supplementary material shows.

The observed electrostatic differences between the seven-bladed WD β-propeller protein subfamilies may imply that the surface electrostatic pattern diverged with the protein function during evolution while the β-propeller fold remained conserved throughout the WD40-repeat family. The RMSD and sequence identity as well as sequence similarity comparisons (Fig. 2) show that the sequence identity between seven-bladed WD β-propeller representative structures is as low as 10%. This is an interesting observation because RMSD between different WD β-propellers is low, in the range from 1 to 3 Å (Fig. 2). While high sequence identity may suggest similarity of function, very low sequence identity or similarity does not rule out similar function, especially if the fold is very conserved. Here our results show that protein function can be distinguished based on the electrostatic properties of protein subfamilies with very low sequence identity, but strikingly similar fold.

### WD propellers exhibit a continuum of functional sites

Studies of the WD40-repeat family reveal that it has multiple binding partners and it has also been suggested that it serves as a scaffold for protein-protein interactions (Paoli, 2001). To investigate this functional divergence further we have analyzed the spatial distribution of the functionally important amino acids by a combination of ET, surface curvature analysis and mapping the experimentally determined functionally important residues on to the protein structures.

**Figure 6:**
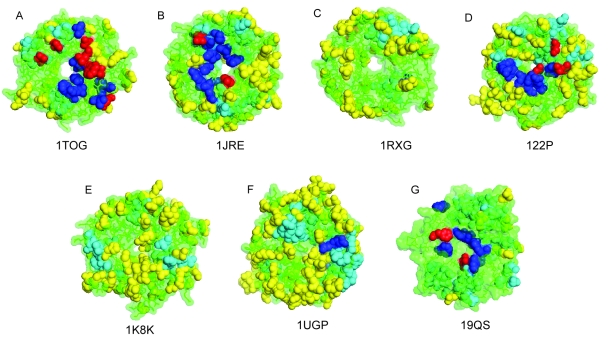
The seven-bladed WD protein families reveal a continuum of functional sites. The residues proposed to be functionally important are mapped on to experimental structures. Blue—residues identified by mutational experiments, Yellow – exposed class-specific residues classified by ET in the trace 10, Red shows the overlap between ET and experimental mutations, Cyan – residues proposed to form cavities on the surface. (A) shows the WD part of G-beta protein (1GOT), (B) Transcriptional repressor Tup1 (1ERJ), (C) Transcriptional repressor Groucho (1GXR), (D) F-box/WD-repeat protein 1 (1P22), (E) Arp2/3 complex (1K8K), (F) Actin interacting protein 1 (1PGU), (G) Ski8p, mRNA degradation regulating protein (1SQ9). All proteins are oriented in an identical fashion.

ET is a powerful tool for prediction of protein functional sites. The method uses a set of aligned sequences and the corresponding phylogenetic tree. Here the evolutionary privileged residues predicted as exposed class-specific in the 10th partition (see Methods) are highlighted on the surface (Fig. 6). These residues may be functionally important, but in order to identify others or confirm these we have additionally applied a program developed by Tsodikov et al. (Tsodikov et al. 2002) to search for regions forming cavities on the surface, because these may also suggest possible binding sockets. It has not been suggested that any of the seven-bladed WD β-propeller families have an enzymatic activity, we therefore analyse the small cavities on the surface as these may suggest possible allosteric interaction sites. The surface residues, creating small cavities that are shown on the representative structures (Fig. 6), suggest potential binding pockets. One of the best ways to characterize the binding areas is obviously experimental. We have done a literature search to find surface residues reported to be involved in interactions in seven-bladed WD β-propellers. The residues experimentally determined to be functionally important in each seven-bladed WD β-propeller subfamily (Table 1), were mapped onto the corresponding structures. Figure 6 shows the continuum of potential functional sites for all seven subfamilies. Although the predictions reveal wide distribution, it appears there is a higher probability for functional interaction sites on the “top” side of the WD β-propeller subfamilies. Previous experimental studies also report that functional residues are located mainly on the “top” interface (Pflugrad et al. 1997; Ford et al. 1998).

The ET method, surface cavities mapping and even the experimental mutations by no means perfectly indicate interaction sites. However, the regions of residues overlap or predicted neighbouring patches are strong indicators of functional importance. The two theoretical techniques in combination with experimental mutagenesis data (where available) suggest that all seven-bladed WD β-propeller subfamilies have multifunctional interfaces, a property also observed in PH domains (Shaw, 1996). Based on our results, we suggest that these residues are likely to be important for protein function: i) G-beta protein subfamily: K89, S98, W99, L117, N119, D186, D228 are experimentally determined functional residues that are also predicted to be functionally important by ET. Unfortunately this overlap is incomplete as can be seen from Table 1. There are experimentally determined functionally important residues not found by evolutionary trace. This trend is seen in most of the subfamilies. S97, D118, G141, T164, G185, A206 are predicted by the ET and surface curvature programs and located next to the residues identified by both experimental mutations and ET ii) Tup1 protein subfamily: Y489, N673 are the experimentally determined functional residues that are also predicted to be functionally important by ET; R652, H671 are predicted by the surface curvature program and located next to residues identified by experimental mutations and ET; Q486, R465, D464, V346, L547 are predicted by the ET and surface curvature programs and located next to residues identified by both experimental mutations and ET; iii) Groucho protein subfamily: H646 is the only residue (of the three possible ones) identified by experimental mutations and ET; iv) F-box/WD-repeat protein subfamily: R285, S325, R474 are identified by experimental mutations and ET; T266, K268, N287, T307, S327 are predicted by the ET and surface curvature programs and located next to residues identified by experimental mutations and ET; G408, R410, N450, E471 are predicted by the ET and surface curvature programs and located next to residues identified by experimental mutations; v) Ski8p, mRNA degradation regulating protein superfamily: F89, R237 are identified by both experimental mutations and ET.

The residues predicted by the ET do not appear to significantly overlap with the experimentally determined functional residues, suggesting that the method may not be as robust as would be hoped. However, this might be due to the fact that WD proteins have a diverse interaction interface and only a limited number of residues important for functional interactions are reported in the literature. This also demonstrates the significance of the results with regards to electrostatic clustering, on a family as functionally diverse as the WD proteins. It is clear that sequence based methods (even those using some structure) such as ET are not able to capture the complexity of the family. We believe, that additional experimental studies of the functional interaction sites might reveal better correspondence between the ET predictions and the experimental data.

It has already been suggested earlier that the WD fold can serve as a scaffold for other protein-protein interactions. Here we support that hypothesis by demonstrating the continuum of potential binding sites on the surface of all analyzed seven-bladed WD subfamilies.

## Conclusions

In this study we have analysed the seven-bladed WD40-repeat protein family a member of the seven-bladed β-propeller fold. We have investigated a variety of characteristics of the experimental structures and the set of homologous models representing seven different seven-bladed WD β-propeller subfamilies that form the family. The models were built in a fully automated manner, to investigate the functional similarities and differences of the seven-bladed WD β-propeller proteins. We have shown that it is possible to classify WD β-propeller protein subfamilies based on the value gained from large scale homology modelling.

We found that the WD40-repeat family is structurally distinct from all other superfamilies forming the seven-bladed β-propeller fold. This difference we believe is due to the specific WD sequence motif, which must lead to distinct structural differences. In fact the sequence identity amongst the WD subfamilies is as low as sequence identity between non WD and other families forming the fold. Our finding allows us to suggest that the WD motif translates into the higher structural similarity and makes the WD40-repeat family distinct in structural as well as sequence terms from other superfamilies of the fold.

We have analysed the surface electrostatics of the seven-bladed WD β-propeller subfamilies and found that it is highly conserved within each subfamily, but differs between subfamilies. The analysis of the surface electrostatics of homology models with sequence identity to the template structure as low as 30% reveals a high degree of electrostatics conservation. The comparison of 166 homology models showed that it is possible to discriminate between the seven-bladed WD β-propeller subfamilies using electrostatics, if the homology models are reasonable. Clustering analysis of the electrostatic results allowed quantification of the differences and similarities between the seven-bladed WD β-propeller subfamiles.

## Supplementary Material

Figure S1 Representative experimental structures for all seven-bladed WD β-propeller superfamilies. The structures shown reveal some variability mostly observed in loops. (A) Galactose Oxidase, (B) Surface layer protein, (C) Nitrous oxide reductase, (D) G-beta protein, (E) Regulator of chromosome condensation RCC1, (F) Clathrin, (G) Integrin, (H) Prolyl oligopeptidase, (I) Tricorn protease, (J) 3-carboxy-cis, cis-mucoante lactonizing enzyme, (K) Putative isomerase YbhE, (L) Sema domain (found in proteins involved in development, tissue regeneration and cancer), (M) Backbone representation of the G-beta protein.

Figure S2 Geometrical characterization of the experimental seven-bladed β-propeller structures. Each sphere represents the middle position on the outer strand of the β-propeller blades. The representative experimental structures from Figure 1 B were superimposed and are each shown in a different colour. Despite some degree of structural variability observed across seven-bladed β-propellers (Fig. 1 in Supplementary Material), these data suggest that the β-propeller blades are spatially conserved across the superfamily.

Table SI Protein sequences used for the electrostatics analysis.
