# Supplementary figures and images for: Electrostatic and Functional Analysis of the Seven-Bladed WD β-Propellers
Source: Evol Bioinform Online. 2008 Jun 13;4:203–16. doi: 10.4137/ebo.s743 (PMC2614187; doi:10.4137/ebo.s743)

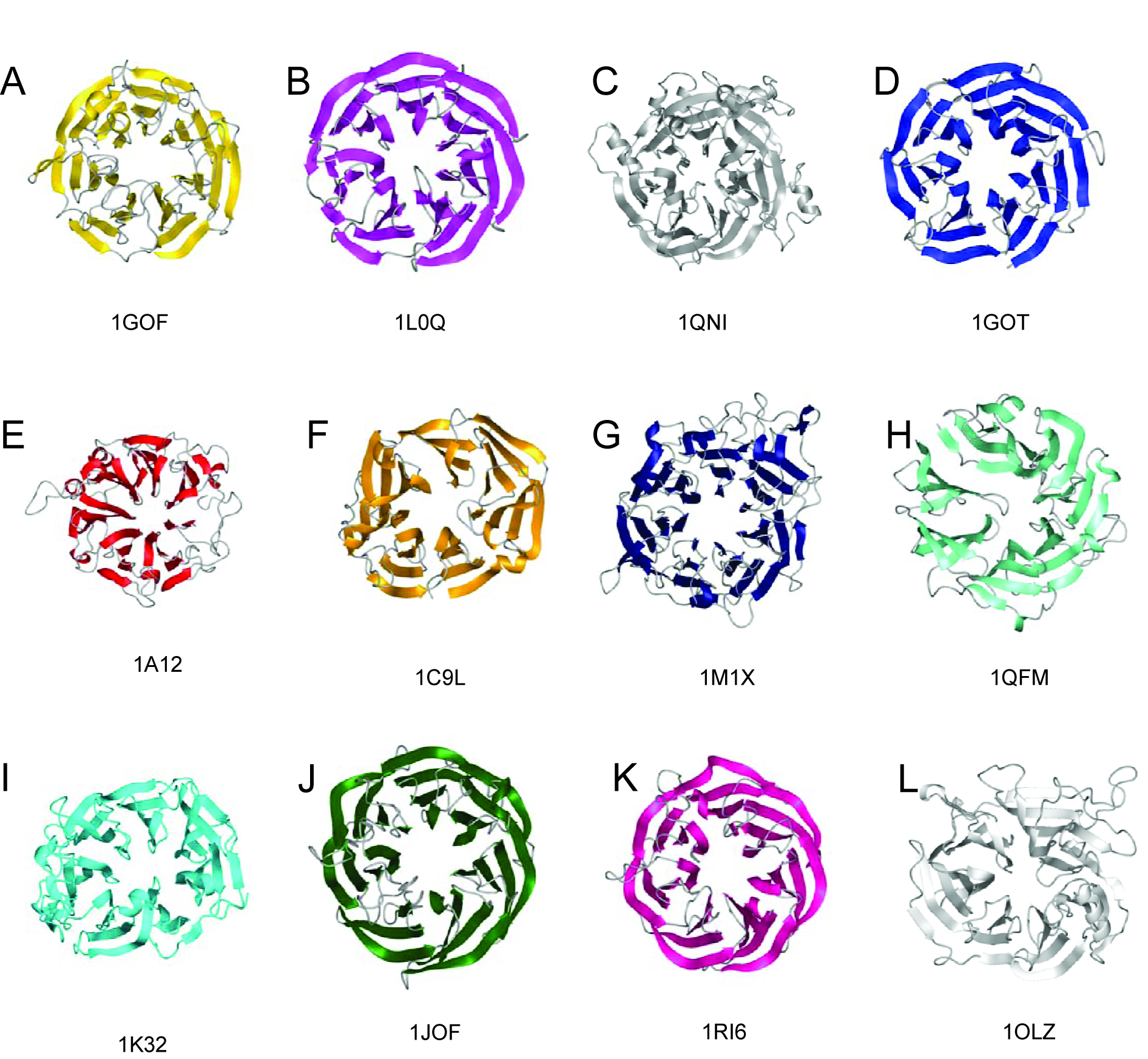

Supplement: Figure S1 — The structures shown reveal some variability mostly observed in loops. (A) Galactose Oxidase, (B) Surface layer protein, (C) Nitrous oxide reductase, (D) G-beta protein, (E) Regulator of chromosome condensation RCC1, (F) Clathrin, (G) Integrin, (H) Prolyl oligopeptidase, (I) Tricorn protease, (J) 3-carboxy-cis, cis-mucoante lactonizing enzyme, (K) Putative isomerase YbhE, (L) Sema domain (found in proteins involved in development, tissue regeneration and cancer), (M) Backbone representation of the G-beta protein. [file ebo-04-203-s01.tif]
