# Supplementary material for: Electrostatic and Functional Analysis of the Seven-Bladed WD β-Propellers
Source: Evol Bioinform Online. 2008 Jun 13;4:203–16. doi: 10.4137/ebo.s743 (PMC2614187; doi:10.4137/ebo.s743)
Supplement: Table SI — Protein sequences used for the electrostatics analysis. [file ebo-04-203-s03.pdf]

Table I. Protein sequences used for the electrostatics analysis

Sequence NCBI ID	Protein name	Subfamily
173067	Tup1	**Tup1**
2245632	Tup1	
4460	Unnamed protein product	
44985561	AGL234Wp	
49524638	Unnamed protein product	
49525018	Unnamed protein product	
49644683	Tup1	
6319926	General repressor of transcription	
9955109	Tup1	
11127727	G-beta	**G-beta**
13591874	G-beta	
15451370	Hypothetical protein	
15451392	Hypothetical protein	
1729803	G-beta	
27597250	G-beta	
27881927	G-beta	
29789261	G-beta	
30583449	G-beta	
34785176	G-beta	
37577053	G-beta	
4139469	G-beta	
47229791	G-beta	
49257618	G-beta	
50752442	G-beta	
53130298	Hypothetical protein	
54261466	G-beta	
54311484	G-beta	
55621538	G-beta	
55732290	G-beta	
57087591	G-beta	
57110000	Predicted to be G-beta protein	
61885523	Similar to guanine nucleotide-binding protein	
62858663	Hypothetical protein	
71872	GTP-binding regulatory protein beta-2 chain	
814	G-beta	
984553	G-beta	
10047159	KIAA1547 protein	**Groucho**
12643289	Transducin-like enhancer protein	
13536985	Groucho-related gene 3 protein	
13879384	Tle3 protein	
1420929	Groucho-related gene 1 protein	
1934855	Groucho 1 protein	
1934857	Groucho 2 protein	
20532417	Transducin-like enhancer protein	
21730272	GrouchoTLE1	

26245355	Groucho	
26245359	Groucho	
26245361	Groucho	
26327207	Unnamed protein product	
26343129	Unnamed protein product	
27469815	TLE3 protein	
28972780	mKIAA1547 protein	
29337235	Transducin-like enhancer protein	
29429207	Transducin-like enhancer protein	
307510	Transducin-like enhancer protein	
307516	Transducin-like enhancer protein	
31543870	Transducin-like enhancer protein	
33991693	Tle3 protein	
34783362	TLE3 protein	
34915994	Transducin-like enhancer protein 4	
35505280	Transducin-like enhancer of split 1	
38372895	Transducin-like enhancer protein 4	
4028902	Groucho-related gene 4 protein	
4033595	Groucho	
45383652	Transducin-like enhancer of split 4	
46391800	Transducin-like enhancer of split 3 splice variant 1	
46391802	Transducin-like enhancer of split 3 splice variant 2	
47225606	Unnamed protein product	
47225892	Unnamed protein product	
4827030	Transducin-like enhancer protein 3	
49022870	mKIAA1261 protein	
50762354	Similar to transducin-like enhancer protein 1	
50925319	TLE4 protein	
54300418	Groucho	
55631783	Hypothetical protein	
55959469	Transducin-like enhancer of split 1	
55959926	Transducin-like enhancer of split 4	
56207263	Transducin-like enhancer of split 1	
56207264	Transducin-like enhancer of split 1	
56207265	Transducin-like enhancer of split 1	
56207266	Transducin-like enhancer of split 1	
57035956	Similar to transducin-like enhancer protein 4	
58384323	WD protein with a variety of functions	
58391041	Hypothetical protein product	
61818431	similar to transducin-like enhancer protein 3	
62649115	Similar to transducin-like enhancer protein 1	
6330948	KIAA1261 protein	
6678361	Transducin-like enhancer protein 3	
68390012	Similar to Groucho2 protein isoform 3	
68390016	Similar to Groucho2 protein isoform 5	
68390018	Similar to Groucho2 protein isoform 6	
68390025	Similar to Groucho2 protein isoform 9	

68390025	Similar to Groucho2 protein isoform 9	
68390032	Similar to Groucho2 protein isoform 12	
68390040	Similar to Groucho2 protein isoform 14	
68390044	Similar to Groucho2 protein isoform 16	
68390048	Similar to Groucho2 protein isoform 18	
68390051	Similar to Groucho2 protein isoform 19	
68390053	Similar to Groucho2 protein isoform 20	
68390055	Similar to Groucho2 protein isoform 21	
68390057	Similar to Groucho2 protein isoform 22	
68390063	Similar to Groucho2 protein isoform 25	
68390191	Similar to Groucho2 protein isoform 3	
68390193	Similar to transducin-like enhancer protein 3	
68390195	Similar to Groucho2 protein isoform 5	
68390197	Similar to Groucho2 protein isoform 6	
68390199	Similar to Groucho2 protein isoform 7	
68390202	Similar to Groucho2 protein isoform 8	
68390204	Similar to Groucho2 protein isoform 9	
68390206	Similar to Groucho2 protein isoform 1	
68390208	Similar to Groucho2 protein isoform 10	
68390210	Similar to Groucho2 protein isoform 11	
68390212	Similar to Groucho2 protein isoform 12	
68390215	Similar to Groucho2 protein isoform 13	
68390217	Similar to Groucho2 protein isoform 14	
68390219	Similar to Groucho2 protein isoform 15	
68390221	Similar to Groucho2 protein isoform 16	
68390223	Similar to Groucho2 protein isoform 17	
68390225	Similar to Groucho2 protein isoform 18	
68390227	Similar to Groucho2 protein isoform 19	
68390229	Similar to Groucho2 protein isoform 20	
68390231	Similar to Groucho2 protein isoform 21	
68399552	Similar to Transducin-like enhancer protein 4	
71052126	TLE4 protein	
7239366	Groucho-related protein 4	
7328109	Hypothetical protein	
8489101	Transducin-like enhancer 3	
9507191	Transducin-like enhancer protein 4	
12963527	Actin related protein 2/3 complex	**Arp2/3 related protein**
13097399	Actin related protein 2/3 complex	
14043135	Actin related protein 2/3 complex	
17943201	Arp2/3 complex	
62460388	Arp2/3 complex	
9506405	Arp2/3 complex	
16117783	Beta-transducin repeat containing protein isoform 1	**F-box/WD-repeat protein**
23956270	F-box and WD-40 domain protein 11	
26006203	mKIAA0696 protein	
3327206	KIAA0696 protein	
33357846	Beta-Trcp1-Skp1-Beta-Catenin Complex	

34870632		
41152004	F-box and WD-40 domain protein 11	
4140718	Beta-transducin repeat containing protein	
4502477	Beta-transducin repeat containing protein isoform 2	
47087275	Hypothetical protein LOC406825	
47210478	Unnamed protein product	
48928046	F-box and WD repeat domain containing 11 isoform B	
48928048	F-box and WD repeat domain containing 11 isoform A	
48928050	F-box and WD repeat domain containing 11 isoform C	
50749709	Similar to F-box/WD-repeat protein 1A	
50924922	Btrc-a protein	
51476320	Hypothetical protein	
5230822	Beta-transducin repeat-containing protein	
53136810	Hypothetical protein	
54648390	Beta-transducin repeat containing protein	
55634331	Similar to beta-transducin repeat containing protein	
55726548	Hypothetical protein	
56207155	F-box and WD-40 domain protein 1B	
56207156	F-box and WD-40 domain protein 1B	
57085197	Similar to F-box and WD-40 domain protein 1B isoform B	
6753210	Beta-transducin repeat containing protein isoform b	
34811520	Yeast Actin Interacting Protein 1 (Aip1)	**Yeast Actin Interacting Protein 1 (Aip1)**
34811521	Yeast Actin Interacting Protein 1 (Aip1)	
6323739	Actin cortical patch component	
44984108	AER439Wp	**Antiviral protein Ski8p**
49526867	Unnamed protein product	
49640203	Unnamed protein product	
58176701	Antiviral protein Ski8p	
6321225	Protein involved in exosome mediated 3' to 5' mRNA degradation	

This table shows the sequences used for the electrostatic analysis.
